# Evaluating standards of care in psoriatic arthritis of the QUANTUM project (qualitative initiative to improve outcomes): results of an accreditation project in Spain

**DOI:** 10.1007/s00296-020-04632-2

**Published:** 2020-06-25

**Authors:** Emilio Ignacio García, Mercedes Guilabert, Rubén Queiro, Irene Carrillo, José Joaquín Mira

**Affiliations:** 1 Universidad de Cádiz, Cádiz, Spain; 2 Calite Research Group, Universidad Miguel Hernández de Elche., Health Psychology Department, Altamira Building, Avenida de la Universidad, s/n, 03202 Elche, Spain; 3 Rheumatology Division, Hospital Universitario Central de Asturias, Oviedo, Spain; 4 The Foundation for the Promotion of Health and Biomedical Research of Valencia Region, FISABIO, Sant Joan d’Alacant, Spain; 5 Alicante-Sant Joan Health District, Alicante, Spain; 6 REDISSEC, Madrid, Spain

**Keywords:** Psoriatic arthritis, Multidisciplinary, Quality assurance, Quality standard

## Abstract

In Spain, the QUANTUM project has been promoted to reduce variability in clinical practice and improve the care and quality of life of people with psoriatic arthritis (PsA) by accrediting PsA units throughout the Spanish national health system. To present the results of this approach which sought to ensure an optimum level of quality for patients with PsA. Descriptive analysis of the self-assessments that the PsA units have carried out assessing their degree of compliance with the quality standards established in the QUANTUM project grouped into four blocks: shortening time to diagnosis; optimizing disease management; improving multidisciplinary collaboration; and improving patient monitoring. A total of 41 PsA units were self-evaluated. They met 64.1% of the defined quality standards. Optimize disease management obtained a higher level of standards compliance (72%) and improve multidisciplinary collaboration the lesser (63.9%). Accessibility to the treatments available for PsA in all hospitals was guaranteed (100%). Appropriate diagnostic equipment is available (97.6%). Compliance with specific quality standards leads to detect actions that should be implemented: quality of life assessment (9.8%), locomotor system assessment (12.2%), physical examination data record (14.6%), periodic cardiovascular risk assessment (17.1%). The QUANTUM project results make it possible to visualise how to care for patients with PsA is being developed in Spain. Problems identified in recent multinational reports are also identified in Spain.

## Introduction

Psoriatic arthritis (PsA) is a chronic, progressive, multiorgan disease [1] common (10–20%) among patients suffering from psoriasis. It presents a relapsing course throughout life, with periods of inactivity and others of inflammation and pain. The EPISER study [2] has established the prevalence of PsA in Spain in 0.58% of the population over 18 years of age.

GRAPPA (Group for Research and Assessment of Psoriasis and Psoriatic Arthritis) [3] has studied worldwide the factors that contribute to a decrease in the quality of care received by patients diagnosed with PsA. The conclusions of this group indicated variable care across health systems, which may result in progression of the disease does not stop, there is a gradual reduction in function, an increase in disability and a poor quality of life of the patients. For example, in Spain it has been observed that around 60% of the medical records did not quantify the joint involvement and in 87% there was no composite joint index and 84% did not have a measure of function [4, 5].

The quality standards proposed by the NEXUS project [6] or the European League Against Rheumatism (EULAR) [7] recommendations have sought to promote integrated care by the multidisciplinary teams that intervene in PsA [8, 9], and they have been oriented towards improving the quality of healthcare received by these patients, establishing a metric and recommendations for achieving optimal quality of care.

Among the areas of improvement that cover the different stages of PsA, the following have been identified: reducing the current delay in diagnosis (this can be up to two years on average due, fundamentally, to the lack of awareness of the disease and the time available for consultations); achieving management of the disease by multidisciplinary teams; increasing accessibility to the use of biologics in the treatment of PsA; and promoting an adequate management of the comorbidities present in patients with PsA.

In Spain, the QUANTUM (quality initiative to improve outcomes) project was initiated in 2017 [10]. It has been promoted within the framework of QUANTUM international and the GRAPPA initiative [3], to adapt to the Spanish public health system this set of quality standards that seek to reduce variability in clinical practice and improve the care and quality of life of people with PsA. To achieve this objective, a set of good patient management criteria have been elaborated that standardize good organizational, diagnostic and therapeutic practices. A total of 15 PsA units have participated.

This set of criteria is the result of consensus work with 15 specialists who agreed on a proposal for quality criteria (who worked in different hospitals), some of which were considered obligatory (18 in total) and, therefore, indispensable for achieving quality care.

The QUANTUM project has promoted the accreditation of PsA units throughout Spain. This paper presents the results of this exercise which sought to ensure an optimum level of quality for patients with PsA.

## Method

The evaluations carried out and included in this study were based on the quality standards established in the QUANTUM project at the national level [11]. This project defined by consensus a set of criteria for optimal quality of care in PsA. The criteria consisted of 59 standards (18 mandatory and 41 non-mandatory standards), grouped into 4 blocks: shortening time to diagnosis (*n* = 6), optimizing disease management (*n* = 26), improving multidisciplinary collaboration (*n* = 9), and improving patient monitoring (*n* = 18). The level of compliance required varies between standards, in some cases the objective level is dichotomous (yes/no), while in others the compliance required ranges from 50 to 100%, with 80% being the most frequent value. According to the QUANTUM project, PsA units must meet more than 70% of the quality standards (including the 18 mandatory ones) to be accredited.

The PsA units that voluntarily participated in this study individually carried out a self-evaluation to subsequently proceed to the accreditation offered by the QUANTUM project. Only aggregated data collected for the accreditation using QUANTUM criteria were used in this study. The self-evaluation procedure was supported by an online application (https://apsquantum.com) that facilitated this evaluation task. Each unit determined the people who were to conduct this self-assessment and for each standard they had to provide evidence to justify the assessment assigned in each standard.

Where individual units did not meet the quality standards there was the option of drawing up an improvement plan, submitting it to external review on a voluntary basis and, after implementing the agreed measures, requesting an external visit to obtain an opinion on their possible accreditation in accordance with the QUANTUM project standards.

## Results

Between July and September 2019, a total of 41 PsA units were self-evaluated (7, 17.1% located in county hospitals with less than 400 beds and 36, 87.8% in university hospitals). These hospitals belonged to a total of 13 autonomous health services.

The PsA units of these hospitals met 64.1% of the defined quality standards, 71.5% of the obligatory standards and 60.9% of the non-compulsory standards. Although the average compliance rate was close to the level required for accreditation, only two (4.9%) of the participating units met the 18 mandatory standards, so the rest would not be accredited at that stage. The units contributed improvement plans in those cases in which the standard was not satisfied, the calendar for the implementation of these plans varying around 12 months. Table 1 shows the degree of compliance achieved overall by the set of PsA units that carried out the self-assessment procedure in each of the blocks. Table 2 shows the quality standards that the majority of PsA units met, along with those in which almost all had problems reaching the reference value of the standard.

**Table 1:** QUANTUM project. Compliance with quality standards in each block

Block	Number of standards	Number of mandatory standards	Total compliance (%)	Compliance with mandatory standards (%)
Shorten time to diagnosis	6	0	71.5	–
Optimize disease management	26	7	72	71
Improve multidisciplinary collaboration	9	2	63.9	58.5
Improve monitoring	18	9	50.2	74.8

**Table 2:** QUANTUM project. Standards met (90%) and not met (20%) by most PsA units in each block

Block	Standards	Compliance (%)
Shorten time to diagnosis	Family residents and primary care physicians will be provided with rotations for rheumatology services	95.1
Optimize disease management	In the pharmacy catalogue of the centre, all the targets authorised for PsA should be accessible at all times	100
	Centres treating patients with PsA should have access to ultrasound and magnetic resonance imaging (MRI)	97.6
	The consultation where attention is paid to patients with PsA must have or have permanent access to computer/scale and microscope	95.1
	Services should participate in and develop exclusive PsA training activities and continuing education programmes in PsA	92.7
	Outpatient consultations where PsA patients are cared for should be accessible and signalled	92.7
	The radiology service of the hospitals where patients with PsA are treated should have a team specifically dedicated to the locomotor system	90.2
Improve multidisciplinary collaboration	The existence of a vaccination protocol in the service and the referral circuit with preventive medicine should be evaluated to update the vaccination calendar in patients with PsA	92.7
Improve monitoring	It should be performed as complementary tests to the patient with PsA, at least in the two consecutive visits of 6 months or more, hemogram and general biochemistry	97.6
	The value of the C-reactive protein (CRP) in two consecutive visits of 6 months or more and of the erythrocyte sedimentation rate (ESR) on an annual basis should be recorded in patients with PsA	95.1
	In PsA patients, the evaluation of their quality of life with the Psoriatic Arthritis Impact of Disease (PsAID) index should be carried out at least once a year	9.8
	In the clinical history of patients with psoriasis of the dermatology service, it should be noted that specific questions about the locomotor system have been asked at least once a year	12.2
	In the clinical history of a patient with PsA, at least one general physical examination containing auscultation, abdominal perimeter, weight and height should be collected annually	14.6
	In PsA patients, a specific cardiovascular risk assessment should be performed at least every 2 years	17.1

Most PsA units had access to ultrasound and magnetic resonance imaging (40, 97.6%). The centres that had a radiology service that assigned equipment specifically to the musculoskeletal system were 37 (90.2%).

The average time of the first consultation and successive consultations, of 30 min and 20 min, respectively, recommended in the QUANTUM project quality standards was met by 19 (46.3%) units.

In total, 25 (61%) units had a specific protocol for the care of PsA patients. In most units (31, 75.6%) the patient was attended by the same rheumatologist in successive consultations.

In 12 (29.3%) of the units there was no referral protocol from dermatology for suspected PsA. Only 17 (41.5%) of the centres held joint clinical sessions between dermatology and rheumatology. And only 20 (48.8%) had joint protocols for diagnosing and treating PsA.

They reported having a referral in each primary care health centre for the pathology of the locomotor system 25 (61%) units. However, for the most part (39, 95.1%), rotations of family medicine residents in rheumatology services were facilitated.

The units with a specific nursing consultation for patients with PsA were 35 (85.4%). Patient education information was only available in 21 (51.2%) PsA units.

Patient satisfaction was regularly assessed in 17 (41.5%) of the self-evaluated units.

Most of the units (36, 87.8%) had participated in at least one clinical trial in the last 5 years and 16 (39%) held at least 3 clinical sessions per year.

Table 3 shows the result of the internal audits of clinical records foreseen in the QUANTUM project.

**Table 3:** QUANTUM project. Results of the review of medical records to verify compliance with quality standards (*N* = 41)

Standard	Compliance *N* (%)	Average (SD)	C.I for the mean (95%)	
			Lower lim	Upper lim
The patient’s medical history must include the date of joint diagnosis, the onset of psoriasis symptoms, and the specific form of involvement of that patient (peripheral, axial, mixed, etc.)	21 (51.2)	76.2 (15.4)	71.5	80.9
In the clinical history of a patient with PsA at least one general physical examination containing auscultation, abdominal perimeter, weight and height should be collected annually	6 (14.6)	48.5 (25.4)	40.8	56.2
In patients with peripheral PsA the Minimal Disease Activity (MDA), Disease Activity in Psoriatic Arthritis (DAPSA) or any other validated global activity index should be used, recording all the sections that make up this index on an annual basis in the clinical history	28 (68.3)	54.2 (28.3)	45.6	62.8
Annually, a lipidic profile and uricemia should be performed on the patient with PsA	33 (80.5)	88.8 (15.3)	84.2	93.4
In patients with PsA a specific cardiovascular risk assessment should be performed at least every 2 years	7 (17.1)	37.6 (31.7)	28.0	47.1
In patients with PsA X-rays of hands/feet, pelvis, chest, and symptomatic joints should be performed and included	27 (65.9)	80.1 (16.6)	75.0	85.1
In patients with PsA with peripheral involvement, X-rays of affected joints should be performed with a minimum periodicity of 3 years	12 (29.3)	68.1 (23.1)	61.1	75.1
In patients with PsA the evaluation of disease with the PsAID index should be carried out with a minimum annual periodicity	4 (9.8)	17.2 (25.4)	9.6	24.9

Only the number of units that met the quality standard is reflected in the table

Table 4 shows an example of an improvement plan. The improvement plans contributed by the units were articulated in the following points: quality standard to which reference was made, the definition of the problem identified, description of the causes that cause it, what effect it causes, what activities are planned to be implemented to solve this problem, specification of who should supervise this implementation and when, and specification of what results are expected to be obtained to determine whether the problem has been resolved.

**Table 4:** Example of an improvement plan

Standard	Improvement plan (example)
In PsA patients, the evaluation of their quality of life with the PsAID index will be carried out with a minimum annual periodicity	*What is the problem?* In PsA patients the success of the therapy is marked by the disease activity of the pathology, so the assessment of the quality of life will be a great guidance at the time of decision making *Why does it happen?* In the absence of a protocol that defines what information should be contained in the medical history of patients with PsA, it is up to the physician to decide *Why address it?* The communication between professionals of this or different levels of care will increase the quality of care. The PsAID scale (Gossec et al., a patient-derived and patient-reported outcome measure for assessing psoriatic arthritis: elaboration and preliminary validation of the Psoriatic Arthritis Impact of Disease (PsAID) questionnaire, a 13 country EULAR initiative. Ann Rheum Dis. 2014; 73: 1012–19) developed by EULAR incorporates more elements, including the emotional distress, to take into account the appropriate time for the assessment of pain and the impact of the disease on quality of life. It will make it possible to identify areas that should be addressed in the clinical management of the patient and in the control of longitudinal form *How do we solve it?* A protocol of compliance with the PsAID index should be established with a minimum annual periodicity, where a value less than 4 is considered an acceptable patient status; or a change in relation to the last application of three points is considered an absolute relevant change *Who will do it and when?* It must be performed by the family doctor or rheumatology specialist at the time of the consultation *What results do we intend to achieve?* Implementation is expected in the first year for 45% of patients, in a second year for 65% and in the third year for 85% of patients

## Discussion

The QUANTUM project results make it possible to visualise how to care for patients with PsA is being delivered in Spain, both in terms of resources and the care process.

To our knowledge, this is the first initiative aimed at the accreditation of specialized units in PsA. No equivalent proposals have been identified in other countries and, at the moment, we do not know of any health system outside Spain that has adapted the QUANTUM project. The initiatives developed to date have focused on some specific aspects to improve the quality of care for patients with PsA. Examples of these improvements include assuring multidisciplinary care [12], the incorporation of patient-reported outcome measures (GRAPPA-OMERACT) [13, 14], introducing structural, process and outcome indicators [6], progress in biological treatments and the evaluation of their results (MERECES study) [15, 16]. However, no approach has been identified that focuses on the clinical management of the PsA units, ensuring the cycle of improvement in both care quality and patient safety. The QUANTUM project considers the PsA unit as a whole, with all the aspects that must be considered in the management of this type of unit, including integrated care, specialized training, systematic recording of data in the clinical history, screening, diagnosis, treatment, and evaluation of clinical results [10]. From this perspective, special attention is paid to the necessary collaboration between professionals and specialties involved in the management of patients with PsA (primary care, rheumatology, dermatology and nursing) [17] and the incorporation of validated clinical and psychosocial measures [13, 14, 16].

The data provided in the analysis of the result of the self-assessments show that some of the problems identified in recent multinational reports [4, 7] are also identified in Spain. In view of these results, care integrated by interdisciplinary teams has not been implemented, the implementation of practice guidelines requires greater effort and monitoring of patients over time is highly variable. The care of patients within the framework of a joint face-to-face model [11] is not implemented in all the self-evaluated units.

The supervision of the course of the patients and the coordination between dermatology and rheumatology derived from these evaluations confirm the trend in the improvement of multidisciplinary care observed in some of the studies [8].

There is a high accessibility to the treatments available for PsA in all hospitals. The training of future professionals is fully implemented, including that of primary care doctors, through the resident rotation system. Sufficient and appropriate diagnostic equipment is available. The implementation of electronic medical records contributes to the unification of the data recording system and patient safety. These results suggest that some of the barriers identified in other studies (such as barriers to care for the co-morbidity suffered by these patients or difficulties in identifying patients suffering from PsA among non-rheumatologists) could have been reduced in those centres that meet the QUANTUM project quality standards, in line with the idea that reflection on organisational aspects, multidisciplinary teamwork or sensitisation of other professionals on PsA through self-evaluation contributes to the improvement of the care received by these patients [11].

Indirectly, this study reaffirms the relevance of the standards included in the QUANTUM project and allows for the corroboration of their usefulness in describing the operation of the PsA units and promoting the achievement of adequate levels of care quality. Self-assessment based on the standards of these quality regulations allows centers to identify their strengths and weaknesses, as well as opportunities for improvement [10].

When interpreting these data, it must be considered that they are the result of self-evaluations of PsA units and, therefore, correspond to centres that have taken a step forward in the organisation of care for this patient profile. Although PsA units are located in centres distributed throughout the entire country and are located in both university and county hospitals of different sizes. It must also be considered that the selection of these units has not been random.

PsA units from 13 of the 17 health services in Spain participated in this study. The 41 units involved in the study represent those that have considered to best meet the accreditation criteria of the total number of units currently in operation in Spain. It is expected that the remaining units will join the project and the accreditation process in the medium term. For this purpose, the Spanish Society for Quality Care (Sociedad Española de Calidad Asistencial, SECA) has committed to disseminate the quality standards in the management of PsA and to conduct a face-to-face audit to verify the degree of compliance with these standards in the hospitals that join the QUANTUM project.

The dynamics of accreditation proposed by the QUANTUM project requires the incorporation of improvements in organizations, procedures and results. The 41 participating units are immersed in this task. Each of them has received an individualized report where they identify the improvement actions to be carried out. The QUANTUM project is based on the methodology of the quality assurance cycle. In Europe, the total annual cost of psoriatic arthritis per patient ranges from US$ 10,924 to US$ 17,050 [18]. Compliance with QUANTUM regulations is expected to lead to a reduction in costs by reducing or eliminating non-quality costs [10].

The main limitation is that the self-assessment process often differs from the accreditation process, and there is less compliance than has been stated. On the other hand, some criteria depend on the rheumatology service, but there are some more general criteria of the centers.

## Conclusions

Standards of care for patients with PsA are variable. The QUANTUM project aims to improve these standards through benchmarking. This is the first report of a national endeavour to audit current practice against the existing standards with a view to changing practice, thus completing the audit cycle and improving compliance.

## Data Availability

The data can be requested from the corresponding author.

## Abbreviations

CRP: C-reactive protein
DAPSA: Disease activity in psoriatic arthritis
ESR: Erythrocyte sedimentation rate
EULAR: European league against rheumatism
GRAPPA: Group for Research and Assessment of Psoriasis and Psoriatic Arthritis
MDA: Minimal disease activity
MRI: Magnetic resonance imaging
PsA: Psoriatic arthritis
PsAID: Psoriatic arthritis impact of disease
